# Satellite-based modelling of potential tsetse (*Glossina pallidipes*) breeding and foraging sites using teneral and non-teneral fly occurrence data

**DOI:** 10.1186/s13071-021-05017-5

**Published:** 2021-09-28

**Authors:** Stella Gachoki, Thomas Groen, Anton Vrieling, Michael Okal, Andrew Skidmore, Daniel Masiga

**Affiliations:** 1 International Centre of Insect Physiology and Ecology (icipe), Nairobi, Kenya; 2 Faculty of Geo-Information Science and Earth Observation (ITC), The University of Twente, Enschede, The Netherlands; 3 Macquarie University, Sydney, Australia

**Keywords:** Epidemiology, Extrapolation, Satellite data, Seasonality, Species distribution modelling, Trypanosomiasis, Tsetse flies, Vector-borne disease

## Abstract

**Background:**

African trypanosomiasis, which is mainly transmitted by tsetse flies (*Glossina* spp.), is a threat to public health and a significant hindrance to animal production. Tools that can reduce tsetse densities and interrupt disease transmission exist, but their large-scale deployment is limited by high implementation costs. This is in part limited by the absence of knowledge of breeding sites and dispersal data, and tools that can predict these in the absence of ground-truthing.

**Methods:**

In Kenya, tsetse collections were carried out in 261 randomized points within Shimba Hills National Reserve (SHNR) and villages up to 5 km from the reserve boundary between 2017 and 2019. Considering their limited dispersal rate, we used in situ observations of newly emerged flies that had not had a blood meal (teneral) as a proxy for active breeding locations. We fitted commonly used species distribution models linking teneral and non-teneral tsetse presence with satellite-derived vegetation cover type fractions, greenness, temperature, and soil texture and moisture indices separately for the wet and dry season. Model performance was assessed with area under curve (AUC) statistics, while the maximum sum of sensitivity and specificity was used to classify suitable breeding or foraging sites.

**Results:**

*Glossina pallidipes* flies were caught in 47% of the 261 traps, with teneral flies accounting for 37% of these traps. Fitted models were more accurate for the teneral flies (AUC = 0.83) as compared to the non-teneral (AUC = 0.73). The probability of teneral fly occurrence increased with woodland fractions but decreased with cropland fractions. During the wet season, the likelihood of teneral flies occurring decreased as silt content increased. Adult tsetse flies were less likely to be trapped in areas with average land surface temperatures below 24 °C. The models predicted that 63% of the potential tsetse breeding area was within the SHNR, but also indicated potential breeding pockets outside the reserve.

**Conclusion:**

Modelling tsetse occurrence data disaggregated by life stages with time series of satellite-derived variables enabled the spatial characterization of potential breeding and foraging sites for *G. pallidipes*. Our models provide insight into tsetse bionomics and aid in characterising tsetse infestations and thus prioritizing control areas.

**Graphical abstract:**

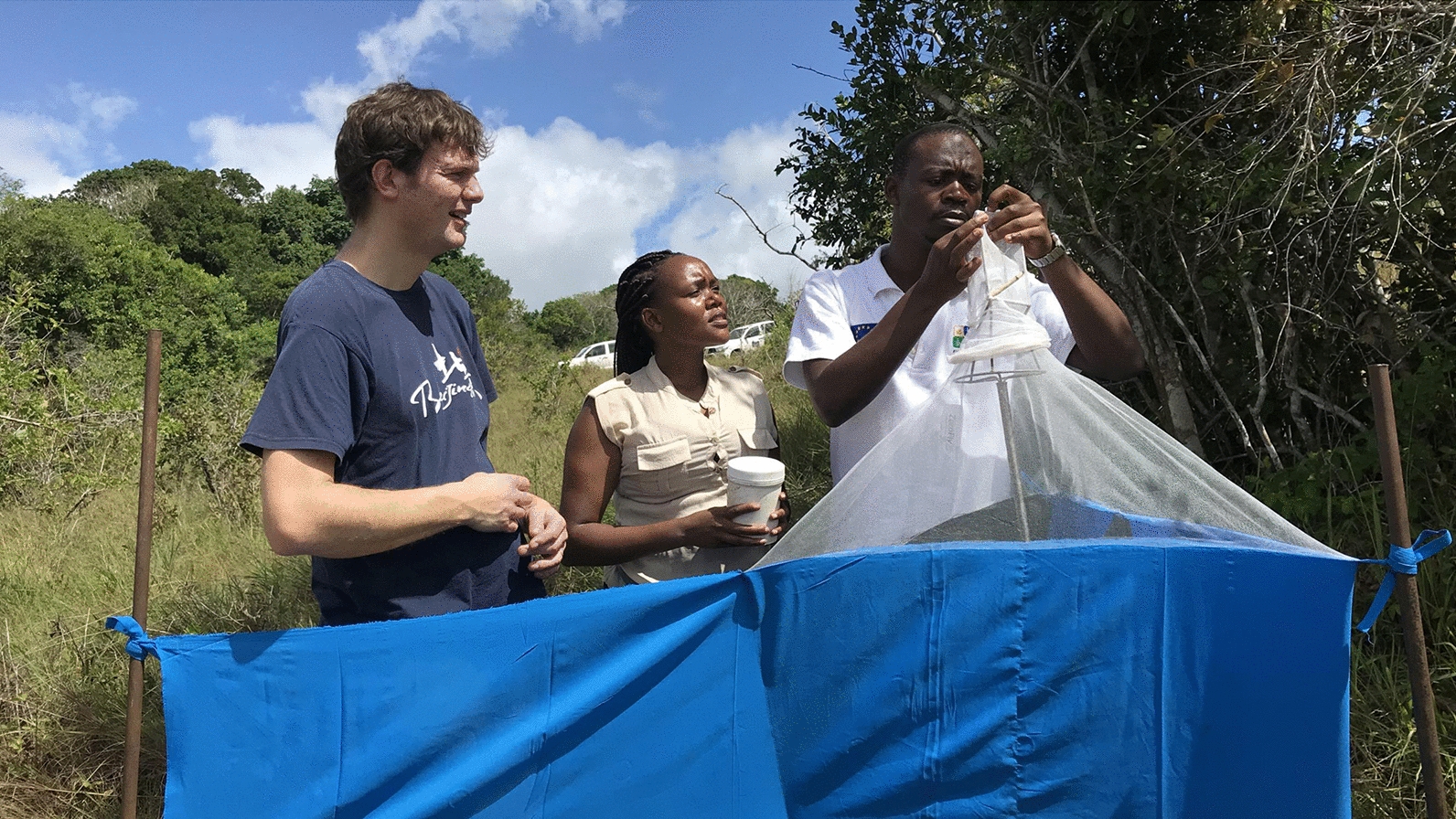

**Supplementary Information:**

The online version contains supplementary material available at 10.1186/s13071-021-05017-5.

## Background

Tsetse flies (*Glossina* spp.) occur only in 38 sub-Saharan Africa (SSA) countries [1]. They are the main vector of trypanosome pathogens that cause animal African trypanosomiasis (AAT) and human African trypanosomiasis (HAT). Although HAT is no longer considered a major public health problem in most of these SSA countries, AAT is still a major constraint in livestock production. In Kenya, the annual economic loss as a result of AAT is projected to be US$ 200 million [2]. There are no vaccines for AAT, and disease control is limited to the use of trypanocidal drugs on infected animals and indirect control measures such as risk mitigation, breeding of trypanotolerant livestock, and vector control [3]. Reducing tsetse numbers in areas in which they occur is the most promising strategy for mitigating AAT. Although tsetse control tools such as the use of odour-baited traps and insecticide-treated targets exist, sustainability is still a problem due to the high costs incurred in their large-scale implementation. Spatially explicit and reliable information on tsetse distribution, particularly their breeding localities, could help guide control by indicating priority areas for strategic targeting.

The occurrence of tsetse flies is determined by the availability of a host to feed on and suitable habitats to breed and rest. In the tsetse breeding cycle, one egg is fertilized by sperm stored in the female’s spermatheca, and then develops through three larval instars inside the ovary before being deposited in an appropriate microhabitat (shaded areas with loosely textured and moist soil with reasonable organic content) where the larva pupates [4, 5]. With sufficient blood meals and a conducive external environment, the development of the larva inside the female fly takes ~ 10 days, while the burrowed pupae take ~ 22–60 days to emerge as a young adult. These young adults that have not had a blood meal (teneral) are unlikely to disperse far from where they emerged, since their flight muscles require 2–3 blood meals and 6–8 days to mature [6]. On the other hand, an adult tsetse fly that has already had a blood meal can fly as far as 1 km away from their habitual grounds to search for a host to feed on [7, 8]. The seasonal changes in the environment affect the distribution of hosts and consequently tsetse feeding habits and localities [9].

Satellite data have been used to predict life-stage-specific suitable habitats for large areas for some insect vectors such as mosquitoes [10–15] by linking satellite-derived environmental variables to species occurrence data. This is yet to be fully exploited for tsetse. Globally, the only existing tsetse fly distribution map predicts the suitability of the general tsetse occurrence at a spatial resolution of 5 × 5 km [1]. The accuracy of this global map is unknown. In some instances, local studies have reported vast differences between the continental map and the actual occurrence of the tsetse fly [16, 17]. Furthermore, the spatial resolution of this global map may be of limited use to guide locally oriented tsetse interventions.

Over the years, identification of tsetse breeding locations has relied on the physical collection of tsetse fly pupae or pupa shells left after birth [18]. Collecting such in situ data is expensive and impractical for large areas. An alternative way to estimate tsetse breeding sites is to capture teneral flies and use their presence as a proxy. However, in situ tsetse fly catches use baited traps that mimic a potential host and can attract tsetse flies from as far as 50 m away [19]. Therefore, although predictive models generally assume that each trap represents a point location, this does not necessarily imply that the environmental conditions at the trap fully represent the preferred habitat. For instance, tsetse fly species such as *Glossina pallidipes* are known to hide in shaded areas and attack host species in open areas. Therefore, the relative abundance of the different land use/land cover (LULC) classes surrounding an area would better represent the *G. pallidipes* occurrence than the actual LULC class at the point location.

We aimed to assess whether the young unfed tsetse flies could be used to model tsetse breeding sites by (1) identifying the environmental variables that explain the occurrence of recently emerged and unfed (teneral) *G. pallidipes* and older flies that have had a blood meal (non-teneral), and (2) using this information to understand, predict, and map the seasonality of the suitable habitats for each life stage using different species distribution modelling techniques. This information will enable more enlightened decision-making and allocation of resources when selecting priority areas for control and piloting of field activities, which is ‘stage 2’ of the Progressive Control Pathway (PCP) for African Trypanosomiasis [20].

## Methods

### Study area

**Fig. 1:**
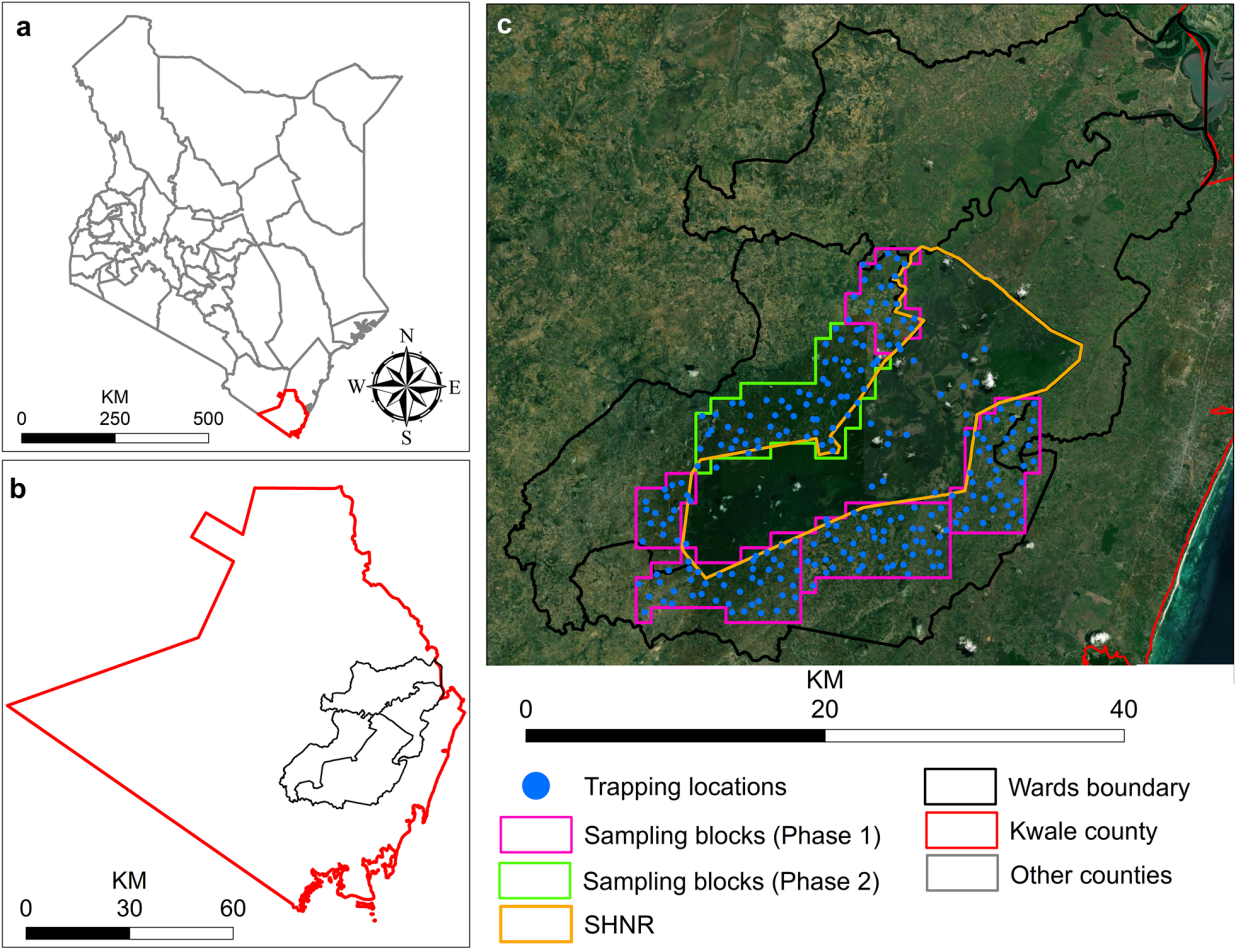
Location of the study area. The grey lines represent the Kenya county boundaries (**a**). The red and the black lines represent the Kwale county boundary and the study area extent, respectively (**b**). The gold lines show SHNR, the blue dots are the tsetse sampling locations, and the pink and green lines are phase 1 and phase 2 sampling blocks (**c**). The background image in (**c**) is the Google Earth image as provided in ArcGIS

The study area covers four administrative wards in Kwale County, Kenya (~ 1173 km^2^, Fig. 1). These wards surround the Shimba Hills National Reserve (SHNR, 192 km^2^), which is managed by the Kenya Wildlife Service (KWS) and is home to diverse wildlife species such as *Loxodonta africana* (African elephant), *Tragelaphus sylvaticus* (bushbuck), *Syncerus caffer* (African buffalo), *Phacochoerus africanus* (warthog), and endangered sable antelope (*Hippotragus niger*). This region is known to have a high tsetse abundance and trypanosomiasis incidence [20–22], with infection rates in cattle often exceeding 30% (Okal et al. unpublished data). The vegetation covers inside SHNR are forests, dense thickets or woodlands, and grasslands with scattered shrubs. The climate is hot and humid with total annual precipitation ranging from 900 to 1500 mm [23]. The communities encircling SHNR cultivate maize, cassava, and tree crops such as cashew nuts, mango, and coconut. The livestock reared in the area are cattle, goats, and chickens. Despite the many years of tsetse control around SHNR, tsetse and AAT is still a major constraint in livestock farming.

### Entomology surveys

Tsetse fly monitoring was done inside and outside of SHNR in two phases between 2017 and 2019. The first phase had 171 monitoring traps outside SHNR (Fig. 1c, pink lines), and the second phase added 60 traps still outside the reserve (Fig. 1c, green lines) and 30 traps inside SHNR. Outside SHNR, sampling was guided by 1 km grids that extended up to 5 km away from the reserve boundary. Inside every grid, one biconical trap baited with cow urine and acetone was installed at a randomly pre-assigned location. Inside SHNR, similar traps were set up, with each land cover having at least six traps. In every period of tsetse monitoring, collections were made every 48 h with four repeats. Since this data collection was only meant for surveillance and not control, the traps were removed after the fourth collection and installed again at the same location during the next period of monitoring.

Individual flies were identified using morphological keys as *G. pallidipes*, *G. austeni*, *G. brevipalpis*, *Stomoxys* spp., *Haematopota* spp., and others. Additionally, for every *Glossina* fly, we recorded the presence of a blood meal (teneral, non-teneral), its sex, and whether the females were pregnant. These parameters were then counted per trap. *Glossina pallidipes* accounted for more than 95% of all the flies captured and were the only species considered in this study.

### Transforming the *G. pallidipes* count data to seasonal occurrence data

**Fig. 2:**
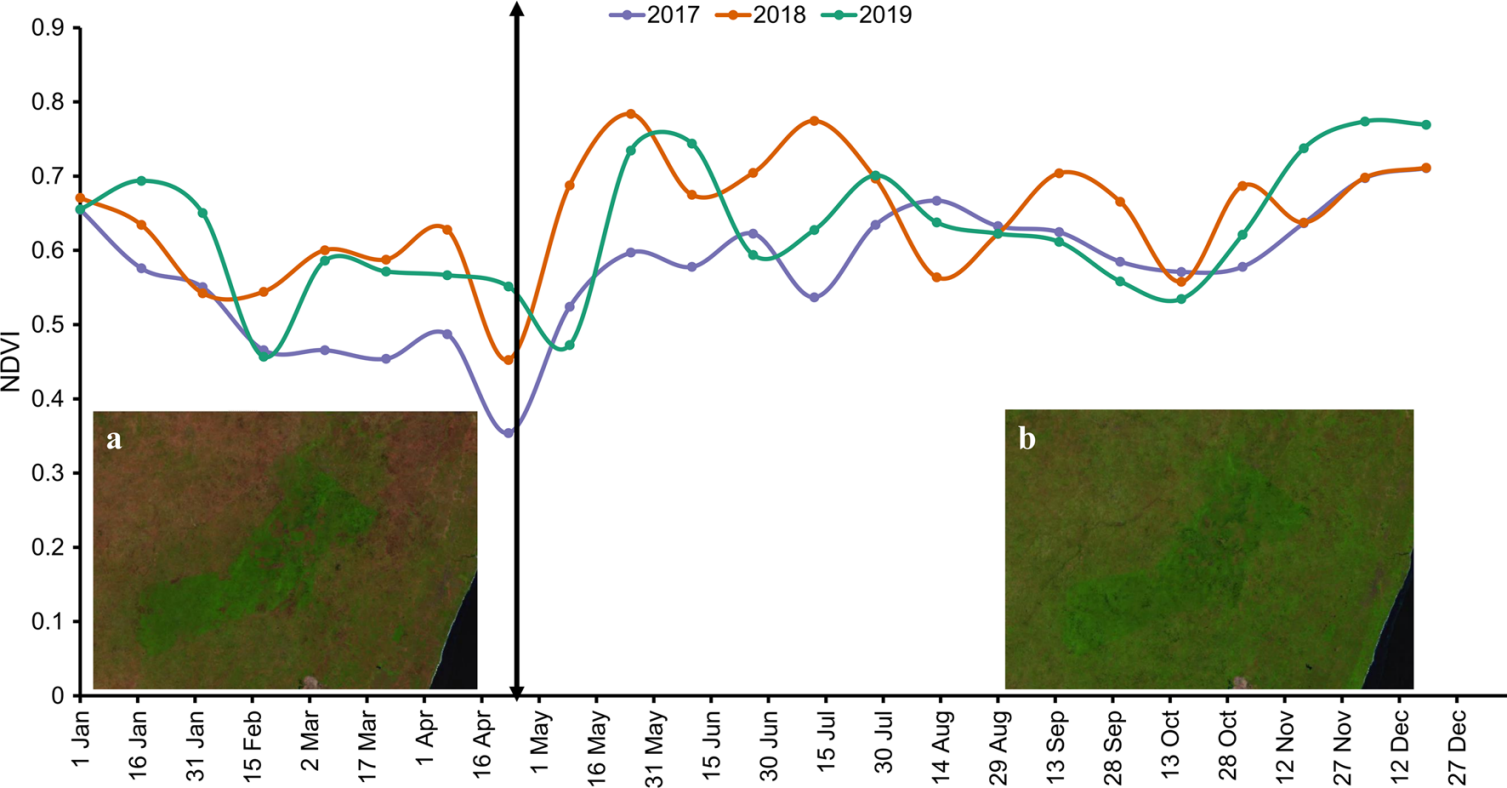
The average 16-day NDVI time series from MODIS as extracted from Climate Engine (https://app.climateengine.org/climateEngine) within the study area for the respective years. **a** and **b** are screenshots of the Sentinel-2 Level 2A median composites (‘B11’, ‘B8’, ‘B4’) for the dry and wet season, respectively

Although the study area has bimodal rainfall, i.e. April–May (long rains) and October–December (short rains), after the start of the long rains, light showers occur during the second dry season (June–September) resulting in high vegetation productivity till the end of the short rains. Since Normalized Difference Vegetation Index (NDVI) variations relate to changes in vegetation cover in semi-arid areas, and *G. pallidipes* depend on the vegetative cover to rest and breed [5], we assumed that the variations in vegetation greenness would influence its distribution. Therefore, instead of using the known rainfall seasons, we delineated two vegetation productivity seasons according to the mean temporal behaviour of the 16-day NDVI obtained from the Moderate Resolution Imaging Spectroradiometer (MODIS) instrument at 500 × 500  m spatial resolution (Fig. 2). The timelines were as follows: 1st January–30th April (dry season), and 1st May–31st December (wet season).

**Fig. 3:**
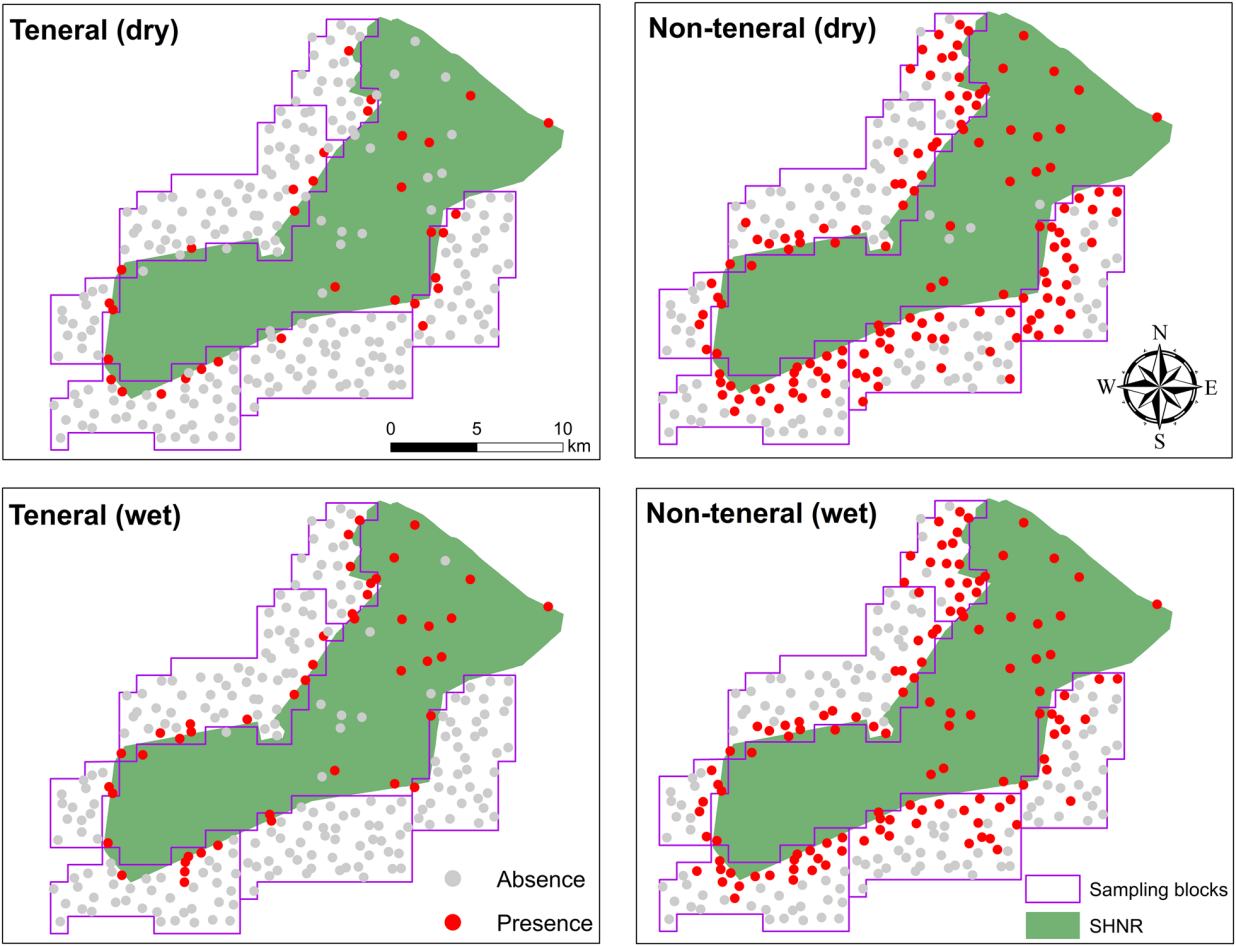
Spatial distribution of the pooled teneral and non-teneral *G. pallidipes* occurrence in and around Shimba Hills National Reserve in the dry and wet season

*Glossina pallidipes* counts per trap were pooled into the dry and wet vegetation seasons using the actual date when the trap was monitored regardless of the year. To transform the tsetse fly count data to binary data, we calculated the flies per trap per day (FTD; divide total tsetse numbers with total monitoring days per trap) for the respective season. Since the shortest monitoring time for one tsetse trap was 8 days, we selected a threshold of 0.125 FTD as the lower limit to consider a catch as a presence (Fig. 3).

### Predictor variables

We used the European Space Agency’s (ESA) Sentinel-2 Level 2A surface reflectance data to generate a land cover layer, and the maximum, minimum, and median NDVI and the Modified Normalized Difference Water Index (MNDWI) at 10 m spatial resolution. These data were accessed and processed using the Google Earth Engine (GEE) platform. Because Sentinel-2 lacks a thermal infrared band, we calculated land surface temperature (LST) using Landsat 8 Operational Land Imager (OLI) surface reflectance data as provided by the National Aeronautics and Space Administration (NASA) in collaboration with the United States Geological Survey (USGS). To derive our predictor variables, for both years and sensors we used a full year of data, i.e., all acquisitions made in 2019. This was done because (1) tsetse data were also pooled into two seasons regardless of the year; (2) our focus is on habitat mapping rather than abundance monitoring; (3) we anticipated that Sentinel-2-derived predictors would show similar spatial patterns in the different years even if the magnitude of the indices might differ; (4) based on MODIS NDVI, 2019 NDVI values were between those of 2017 and 2018 (Additional File 1: Figure S1); and (5) Sentinel-2 surface reflectance data for 2019 were readily available in GEE, which was not the case for previous years. Other variables that were generated include silt percentage (from soil texture analysis data collected at every trapping location), Topographic Wetness Index (TWI), and slope. All variables were resampled to 10 m to match the Sentinel-2 finer resolution. Table 1 summarizes the rationale behind the choice of each variable, while the detailed description of how each variable was extracted is described below.

**Table 1:** Resampled 10 m spatial resolution predictor variables that were used to relate to the presence of teneral and non-teneral *G. pallidipes*

Variable	Rationale/hypothesis
Land cover fraction	Tsetse flies require cool and shaded areas to rest and breed [6]. We hypothesized that areas with high fractions of woodland would exhibit a positive relationship with the presence of the fly, while areas with high fractions of croplands or bare land would have a negative correlation
NDVI	NDVI is a measure of green vegetation abundance. Unlike land cover, which is usually more static, NDVI varies within the year, allowing for a clearer distinction between seasons. NDVI has also been used previously as an indicator of the tsetse ecological niche [57], and based on that study we expect greater abundance for higher levels of NDVI
MNDWI	MNDWI is a spectral index that has been used to delineate open water areas from satellite imagery [58]. Moist environments are crucial for larval survival [6], but areas with too much water can result in the drowning of the larva. We hypothesized that areas with high MNDWI values would result in low teneral fly suitability
LST	LST is the temperature at the top of the canopy retrieved using satellite imagery [24], and it is commonly used as an indicator for air temperature. Several studies have assessed its influence on tsetse distribution, relative abundance, infection rates, and mortality rates [16, 59, 60]. Very high or low temperatures negatively affect the survival of *G. pallidipes* [61]. We hypothesized that areas having temperatures below 20 °C and above 28 °C [5] would result in a lower probability for tsetse presence for both life stages
Silt %	Soils with a large proportion of silt have poor water infiltration [62]. We hypothesized that an increase in silt content in the wet season would result in fewer teneral flies. We assumed that in the wet season, silty soils are more likely to hold high water levels, reducing the probability of burrowed pupae emerging
TWI	TWI quantifies how the land structure controls the hydrological process and has been used to delineate areas that are prone to flooding [63]. Areas with high water retention can cause the drowning of pupae, decreasing their survival rate. We hypothesized that areas with higher TWI values would be negatively associated with the presence of the teneral flies
Slope	Steep areas are vulnerable to soil erosion, while extremely flat areas can be vulnerable to flooding. Whilst erosion or water runoff is also affected by other factors like rainfall intensity [64], we hypothesized that very steep slopes would correlate negatively with the occurrence of teneral flies

### Land cover fractions

We used Sentinel-2 Level 2A data acquired in 2019 and in situ geo-located observations to generate six land cover classes (Table 2).

**Table 2:** Land cover classes that were generated

Land cover class	Description
Forest	Tall high-density trees (> 20 m) with no understory cover
Woodlands	Dense short (< 20 m) thickets
Grasslands	Open grasslands with scattered shrubs and tree crops
Croplands/bare lands	Food crops such as maize, cowpeas, green peas, etc
Settlements	Built-up or developed areas
Water	Water bodies and wetlands

The GPS Essentials (http://www.gpsessentials.com/) Android app was used to collect 140 ground reference data points. We overlaid these point data on high-resolution imagery in Google Earth, and using visual image interpretation we added more points, ensuring that each class had more than 100 points. For every Sentinel-2 Level 2A image, we applied a map function in GEE that used the scene classification layer (part of the Level 2A product) to mask out pixels that had clouds, cloud shadows, and no data. We then generated the seasonal median image composite, which retained the median pixel from the observations of that pixel (within the respective season, Fig. 2), for Sentinel-2 spectral bands ‘B2’, ‘B3’, ‘B4’, ‘B5’, ‘B6’, ‘B7’, ‘B8A’, ‘B11’, ‘B12’, and in addition NDVI, Enhanced Vegetation Index (EVI), and MNDWI indices. The reference data were divided into 70% for training and 30% for testing, and a random forest classifier with 500 trees was applied to the image composite. Table 3 shows the confusion matrix of the land cover classification.

**Table 3:** Land cover test error matrix for the dry and the wet seasons. The blanks cells are zero (0)

	Predicted class					
	F	WD	G	C	S	W
Dry season						
Forest (F)	818	16				
Woodlands (WD)	13	344				
Grasslands (G)			74	1		
Croplands/bare lands (C)			22	63		
Settlements (S)			3	2	904	
Water (W)						720
Wet season						
Forest (F)	812	22				
Woodlands (WD)	58	299				
Grasslands (G)			75			
Croplands/bare lands (C)			23	62		
Settlements (S)				4	905	
Water (W)					10	710

Instead of just evaluating the land cover class at the trap location, we calculated the relative abundance of the different land covers surrounding each pixel (10 m) using a moving window. Within the moving window, the percentage of a specific land cover class surrounding the 10 m pixel was calculated and assigned to the centre pixel. The resulting layer is still at 10 m resolution, but now provides for each pixel information about land cover abundance in its surroundings. This larger spatial configuration is considered important for tsetse fly occurrence, given its range of movements to search for shelter and food. Several moving windows were generated (110 × 110 m, 210 × 210 m, 510 × 510 m, 1010 × 1010 m), and each was tried separately to model the presence of the teneral and non-teneral *G. pallidipes* using the generalized linear model (GLM) with stepwise backward regression. After applying the *step* function (as embedded in the R statistical software) with the land cover fractions generated from all four kernels separately, only the fractions that were calculated using the 1010 × 1010 m window were selected as variables having a significant influence on the occurrence of both life stages. The better performance of 1010 × 1010 m fractions could be attributed to the fact that a tsetse trap mimics a potential host that tsetse can feed on, and a tsetse fly can travel up to 1 km away from their home territory in search of such a host [7].

### Spectral indices

In GEE, we extracted the seasonal (Fig. 2) minimum, maximum, and median composite of NDVI (near-infrared – red/near-infrared + red) and MNDWI (green – shortwave infrared 1/green + shortwave infrared 1) from the cloud-masked Sentinel-2 Level 2A images acquired in 2019. The rationale to include these composites was to account for intra-annual variability of the spectral indices. The corresponding variability in greenness and wetness conditions (Table 1) may be relevant for the distribution of the teneral and non-teneral *G. pallidipes*.

### LST

LST is the temperature that is estimated using satellite imagery and represents the top of the canopy [24]. In this study, the thermal, near-infrared, and red spectral bands from the Landsat-8 OLI were used to estimate LST. In GEE, we used the pixel quality layer (generated by the CFMask algorithm) to mask out clouds and cloud shadows pixels in each of the 18 scenes acquired in 2019 (Worldwide Reference System-2 [WRS-2] row 63, path 166). Subsequently, we followed the procedures described in Jiménez-Muñoz et al. [25] to retrieve LST based on the brightness temperature derived from the thermal band and a fractional green cover estimated from NDVI. The seasonal minimum, maximum, and median LST image composites were generated and resampled to 10 m spatial resolution for further analysis.

### TWI

TWI is a function of slope and flow accumulation and allows for a spatial description of relative soil moisture levels based on water flow. Sørensen et al. [26] give a detailed overview of TWI calculation methods and indicated that the estimation of flow accumulation constitutes a major difference between methods. We used the 30 m Shuttle Radar Topography Mission (SRTM) to estimate the slope and the flow accumulation in ArcMap. TWI was then estimated from the natural logarithm (ln) ratio of the slope and flow accumulation [27]. The result was resampled to 10 m spatial resolution.

### Soil sampling and silt percentage estimation

**Fig. 4:**
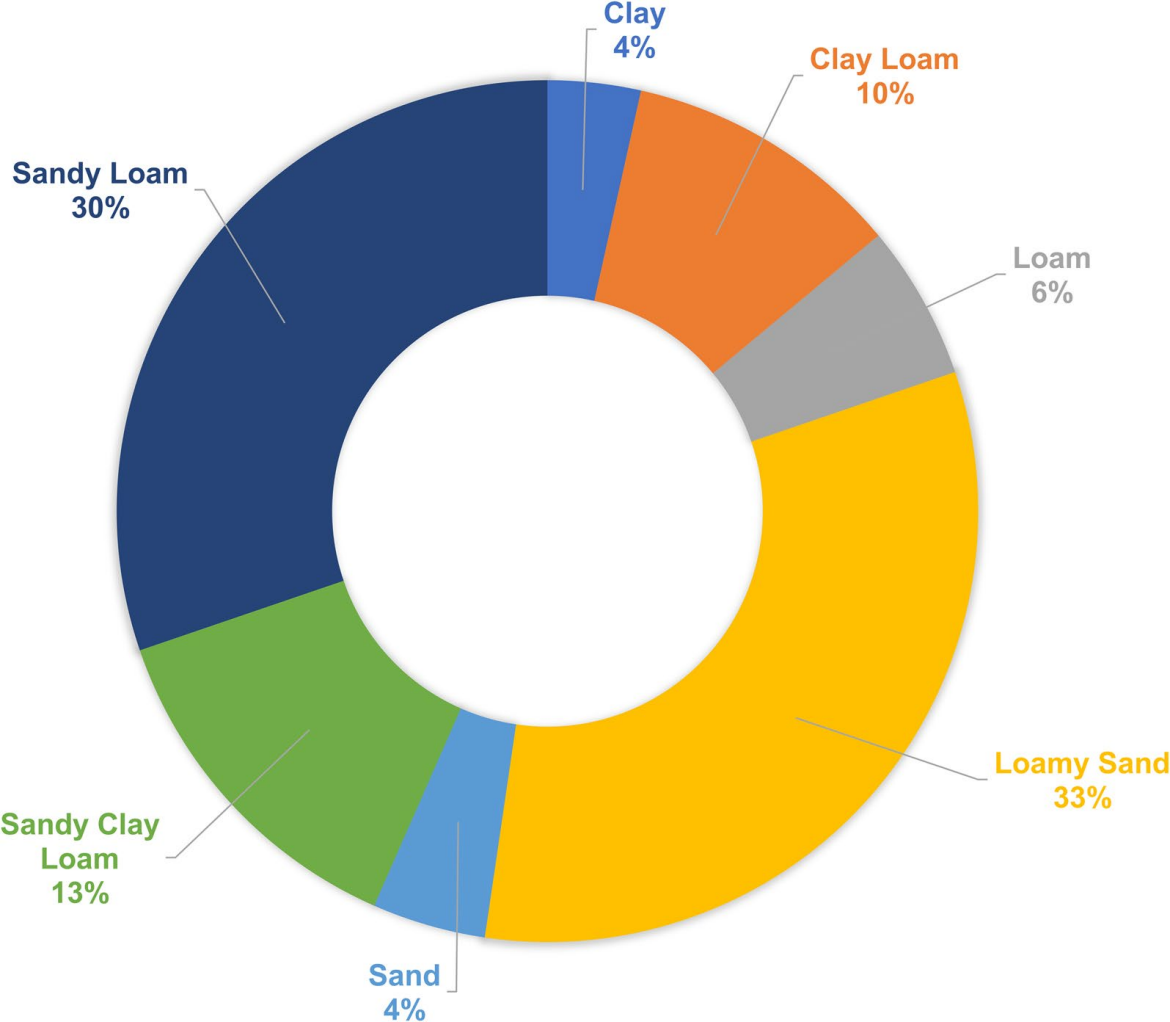
The soil texture type distribution around the Shimba Hills National Reserve (SHNR)

Soil samples were collected within a 1 m square of every tsetse fly trapping location and stored in a plastic container. Soil texture classes were estimated from these samples following the method described in Salley et al. [28]; this constitutes applying water to the sample and evaluating whether it allows for making specific forms. In this way, eight different soil texture types were defined, including clay, clay loam, loam, loamy sand, sand, sandy clay loam, sandy loam, and silt loam. Each soil type was assigned the sand, silt, and clay content ranges as guided by the Food and Agriculture Organization (FAO, Fig. 4). We preferred generating a silt content layer because a future objective is to transfer these models to other regions in Kenya that have more silty than sandy and clayey soils. We used co-kriging in ArcMap to interpolate the field estimated silt content together with the Modified Soil-Adjusted Vegetation Index (MSAVI2), TWI, and slope as covariates, since previous literature had mentioned their importance in representing soil texture variability [28–30].

### Multicollinearity

Before fitting species distribution models, multicollinearity between the environmental variables was assessed using the variance inflation factor (VIF). In a stepwise manner, variables with high VIF values that were considered less relevant to *G. pallidipes* occurrence (based on literature) were excluded until all remaining variables had a VIF value < 10 [31].

### Species distribution modelling (SDM)

Multiple SDM techniques exist with varying strengths and weaknesses, and thus choosing a specific technique can be subjective for a given situation [32]. Therefore, we applied one simple GLM model with backward stepwise regression (GLM*; [33]), two machine learning modelling techniques (boosted regression trees—BRT [34]; and random forest—RF [35]), and one modelling technique that is capable of dealing with collinear data explicitly (GLM with partial least squares regression—GLM-PLSR; [36]) to test whether the most important variables for the occurrence of teneral and non-teneral flies are robustly identified by all four strategies. BRT and RF are machine learning techniques that are designed to learn the model structure from the data, and thus no prior assumptions are needed other than ensuring that the independent variables are uncorrelated. The GLM-based techniques require an additional selection of independent variables that explain as much variation as possible in the response variable [37]. For this study, we used two variable selection techniques for the GLM technique. The first was a backward stepwise regression, which selects the important uncorrelated variables based on the lowest Akaike information criterion (AIC) value. This was performed using the *step* function embedded in the *stats* package in R programming [38]. We then used the *vi* function in the VIP package to confirm whether the retained variables from the *step* function had a variable importance score of > 1 [39]. The second technique was PLSR, which accommodates collinear and correlated variables and uses scores and loadings (a measure of how strongly each variable influences the component) to select variables [40]. A PLSR decomposes the response and explanatory variables into independent components, where the first component explains most variation, and subsequent components explain a decreasing amount of the remaining variation. PLSR models can be tuned to avoid overfitting by selecting the correct number of components to include [41]. In this case, we used the *cv.plsRglm* function to determine the optimal number of components [36]. Biplots were used to assess the set of variables that explained similar variation in the total dataset [36] out of which the most relevant to *G. pallidipes* occurrence was chosen.

We then used the *sdm* package to fit the two GLM models (i.e., with variables selected using *step* function and those selected using PLSR), BRT, RF model, with the last two using all the uncorrelated variables [42]. We determined the relative importance of the variables for the occurrence of the teneral and non-teneral *G. pallidipes* using the *getVarImp* function from the *sdm* package, which considers two metrics, i.e., AUC and the coefficient of determination (*R*^2^). The AUC indicates how well a model can discriminate between areas that are suitable and areas that are not suitable, and ranges between 0.5 and 1. We also reported the true skill statistic (TSS) which indicates the overall classification accuracy of a presence–absence model, provided a certain threshold is used to classify predicted occurrence probabilities into suitable and unsuitable areas. The maximum sum of the sensitivity and specificity was used as an optimal threshold for this [42–44]. We used the *rcurve* function from the *sdm* package to establish the shape of the response of the species to environmental conditions.

## Results

### Selection of predictor variables

**Fig. 5:**
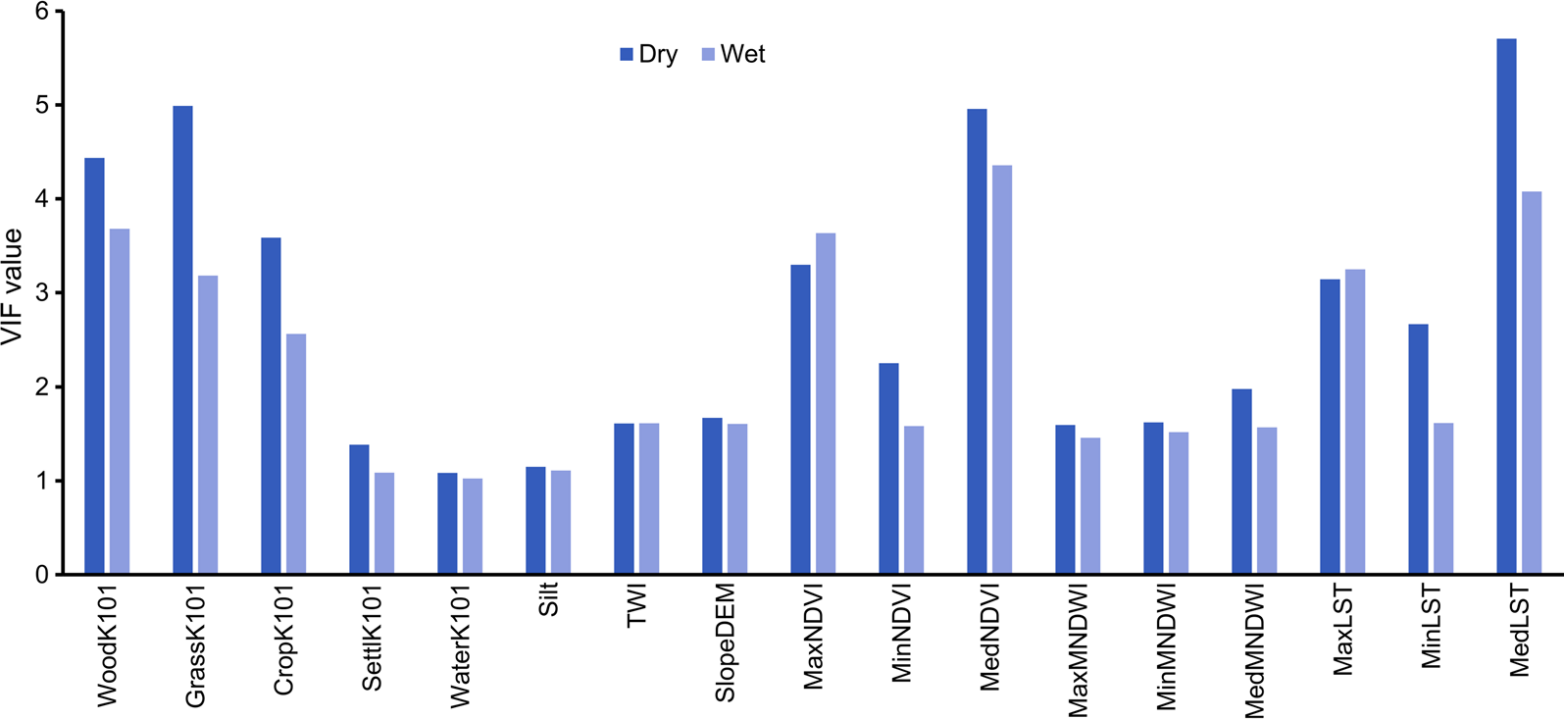
The retained environmental variables that had a VIF value of < 10 after conducting the multicollinearity analysis

**Fig. 6:**
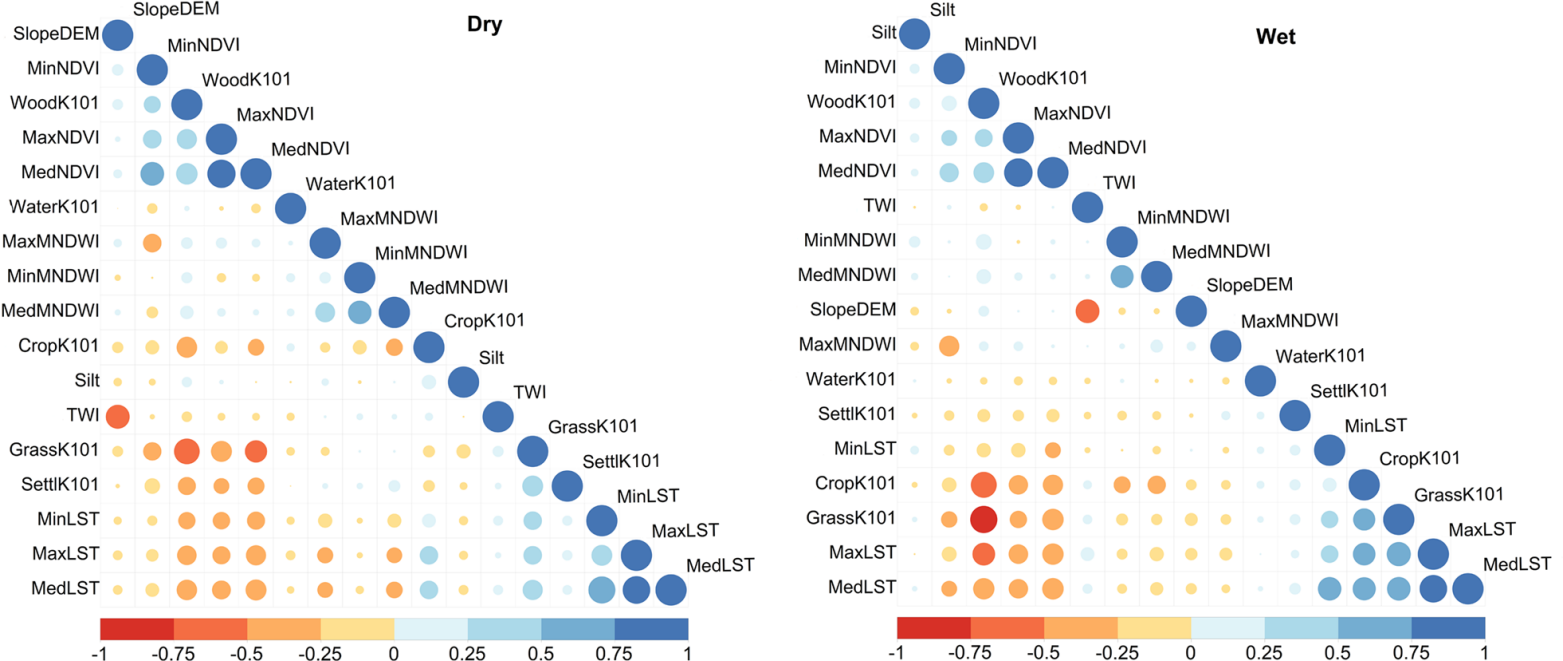
The correlation matrix of the retained variables. Blue colours represent positive collinearity, and red colours negative collinearity. The symbol size represents the strength of the correlation

The retained relevant environmental variables had VIF values below 10 (Fig. 5), and pairwise correlation values were below 0.7 (Fig. 6).

The optimal components for the PLSR models were one and two for the teneral and non-teneral flies in the dry season and two and three for teneral and non-teneral flies in the wet season, respectively. Since biplots cannot be generated for a single component, for the dry season teneral case we included only the variables that had loading values of ≥ 0.4 (Table 4, variables with ^a^) [45].

**Table 4:** PLSR loadings for the teneral *G. pallidipes* during the dry season

Variables	Component_1
ForK101	0.3
WoodK101^a^	0.4
GrassK101	−0.2
CropK101^a^	−0.4
SettlK101	0.0
WaterK101	0.0
Silt	0.0
TWI	−0.1
SlopeDEM	0.2
MaxNDVI	0.3
MinNDVI	0.2
MedNDVI	0.3
MaxMNDW	0.2
MinMNDWII	0.1
MedMNDWI	0.2
MaxLST ^a^	−0.4
MinLST	−0.3
MedLST ^a^	−0.4

^a^The environmental variables that were chosen for further modelling

**Fig. 7:**
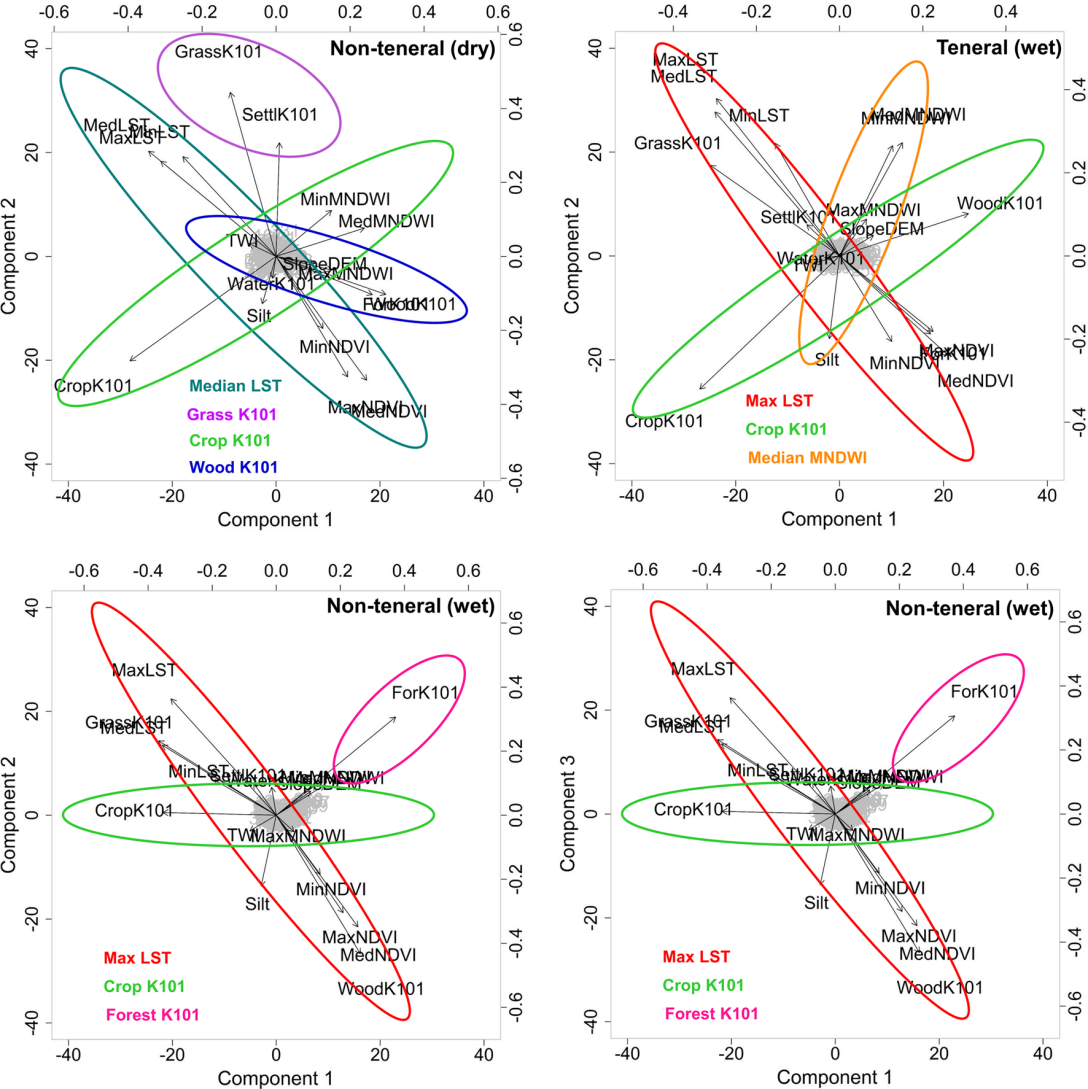
The PLSR components as visualized using biplots. The ovals refer to a set of variables that explain the same amount of variation in the total dataset. The text with the same colour as the ovals indicates the variable chosen to be used in the final model

For the other life stages, biplots were used to visually assess the set of variables that explained the highest amount of variations in the total datasets. From each coloured oval (Fig. 7), a single variable with the longest arrow was chosen for further modelling. Despite the difference in the number of components, cropland fractions were a common variable for both life stages and across seasons.

### Species distribution modelling

**Fig. 8:**
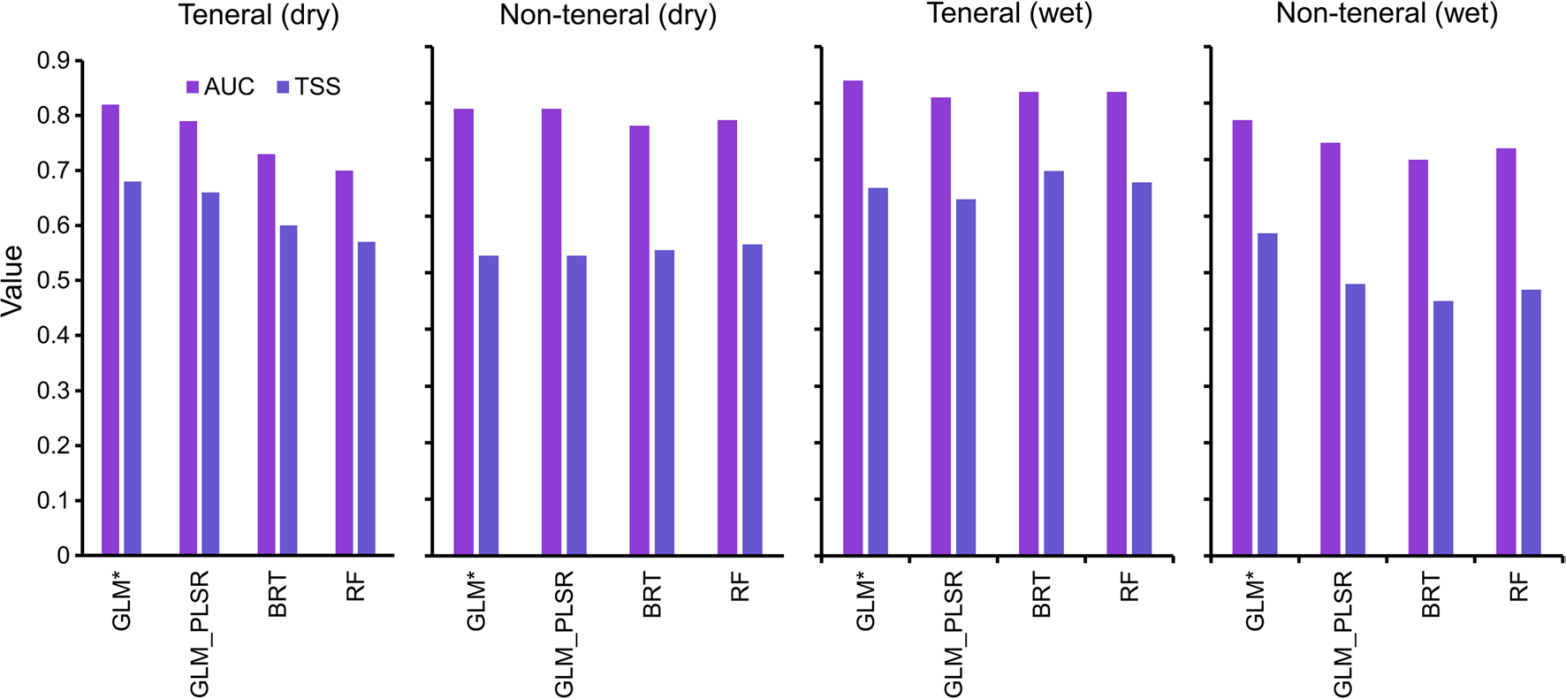
GLM, BRT, and RF model evaluation statistics for the prediction of the teneral and non-teneral *G. pallidipes* occurrence

The AUC for all the models was > 0.7 (Fig. 8). The GLM model with the backward stepwise regression had higher AUC values than all other modelling methods used in both seasons and life stages. For the teneral flies in the wet season and the non-teneral flies in the dry season, the modelling technique used did not influence the AUC values, since all the four models had more or less the same AUC values of ~ 0.83 and ~ 0.78, respectively. For the teneral flies in the dry season and non-teneral flies in the wet season, the AUC values varied between the modelling method used with the latter having the lowest AUC values (~ 0.7).

**Fig. 9:**
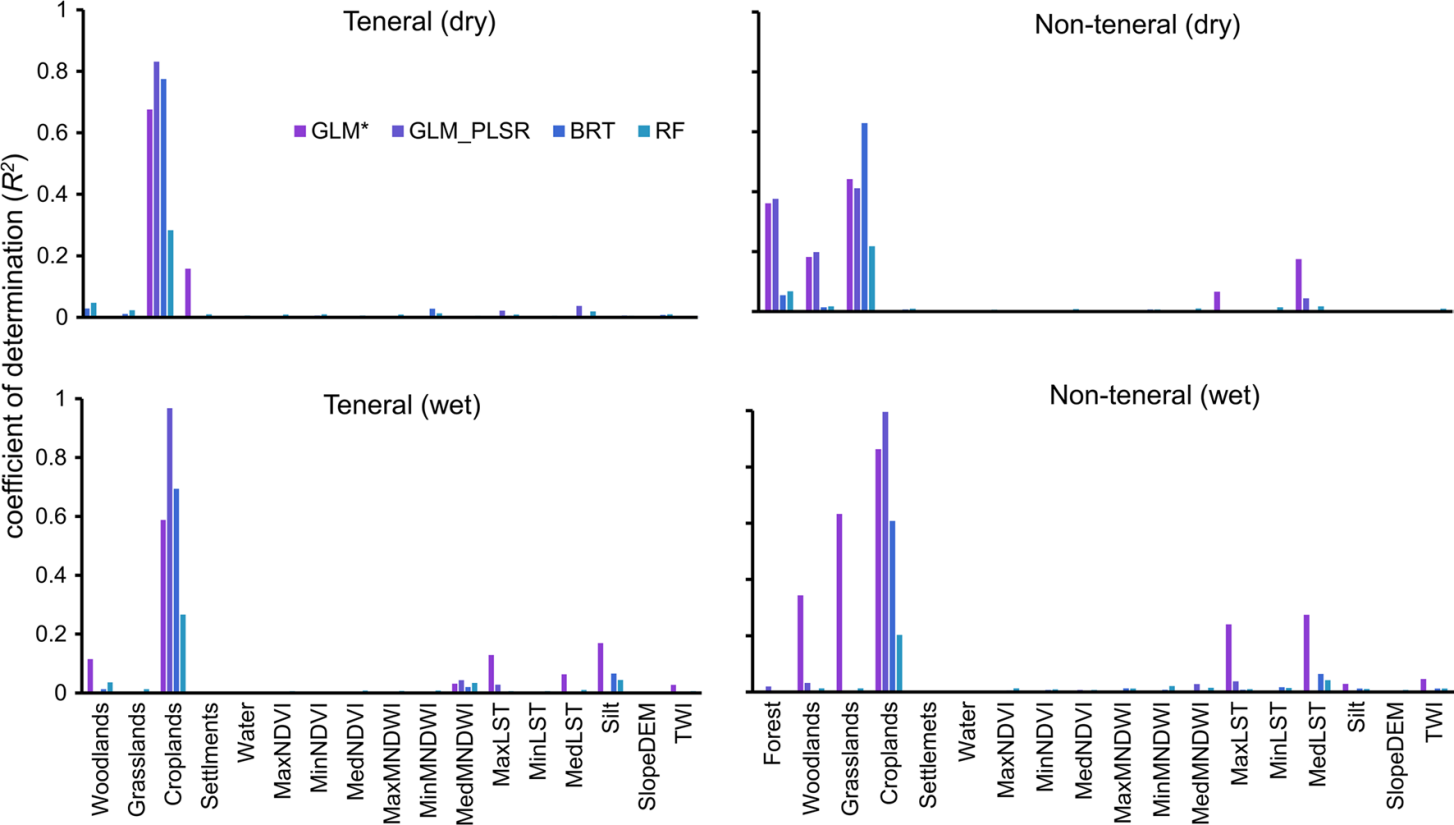
Importance of individual variables for the prediction of the suitability of the teneral and non-teneral *G. pallidipes* using four modelling techniques

**Fig. 10:**
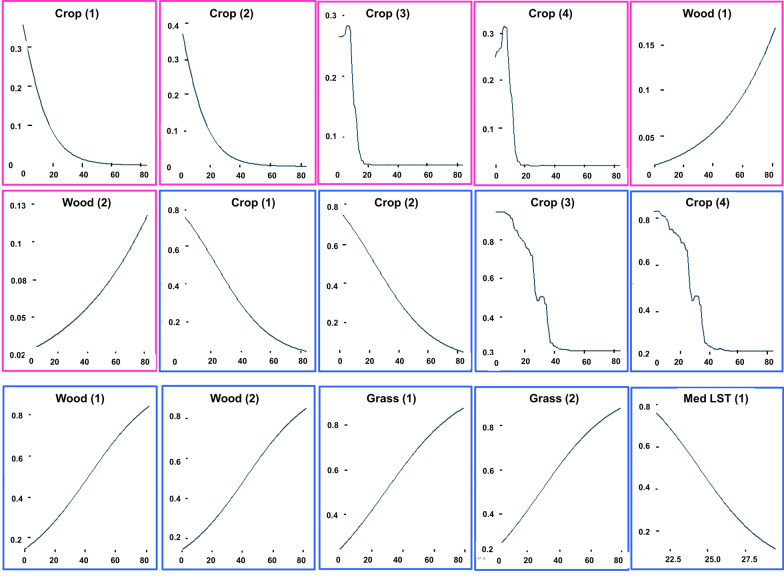
Response (*y*-axis) curves for variables (*x*-axis) having > 0.1 *R*^2^ in the dry season. The panels with the pink boundary refer to the teneral flies, while the ones with the blue boundary are the non-teneral flies. The numbers (1), (2), (3), and (4) at the end of the variable names represent the variable’s response as modelled using the GLM*, GLM-PLSR, BRT, and RF modelling techniques, respectively. The blank/clear cells mean that the variable had *R*^2^ values of < 0.1

**Fig. 11:**
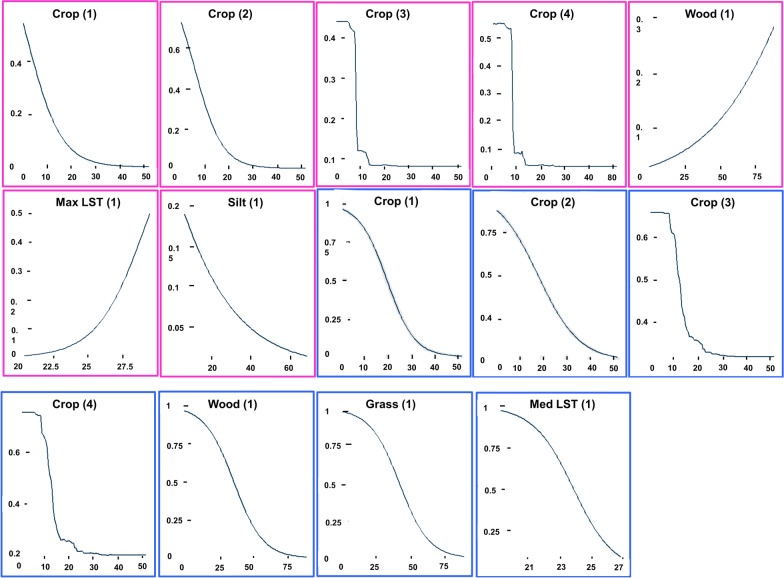
Response curves (*y*-axis) for variables (*x*-axis) having > 0.1 *R*^2^ in the wet season. The panels with the pink boundary refer to the teneral flies, while the ones with the blue boundary are the non-teneral flies. The numbers (1), (2), (3), and (4) at the end of the variable names represent the variable’s response as modelled using the GLM*, GLM-PLSR, BRT, and RF modelling techniques, respectively. The blank/clear cells mean that the variable had *R*^2^ values of < 0.1

Various variables were important for modelling both *G. pallidipes* life stages suitability (Fig. 9). Cropland and woodland fractions were important elements for modelling both life phases and in both seasons. Other factors were only essential for a single life stage, e.g., grassland fractions and median LST were only important for the non-teneral flies, while silt content and maximum LST were essential for the presence of teneral flies (Table 5). The direction and the shape of the relationship between these variables and the presence of *G. pallidipes* varied between life stages and in some instances between seasons for the same life stage (Table 5, Figs. 10 and 11).

**Table 5:** Variables that had *R*^2^ > 0.1. + indicate a positive relationship, while − indicates a negative relationship. The blank/clear cells mean that the variables had *R*^2^ values < 0.1

Predictor variable	Season (D = dry, W = wet)	Teneral				Non-teneral			
		GLM*	GLM-PLSR	BRT	RF	GLM*	GLM-PLSR	BRT	RF
Woodlands	D	**+**	**+**			**+**			
	W	**+**	**+**			**−**			
Croplands	D	**−**	**−**	**−**	**−**	**−**	**−**	**−**	**−**
	W	**−**	**−**	**−**	**−**	**−**	**−**	**−**	**−**
Grasslands	D					**+**			
	W					**−**			
MedLST	D					**−**			
	W					**−**			
MaxLST	W	**+**							
Silt	W	**−**

Cropland fraction correlated negatively with the presence of both life stages, indicating that *G. pallidipes* are less likely to occur in areas that have human-induced changes because of farming. Woodland fraction correlated positively with the presence of teneral flies in both seasons, which may be explained by tsetse’s need for shaded areas for breeding. While the non-teneral flies had a negative relationship with grassland and woodland fractions during the wet season, the relationship was positive in the dry season. This could mean that the woodland fraction and the scattered shrubs/tree crops provided shaded areas for the adult flies to rest in the dry season when temperatures were high.

Teneral flies correlated positively with maximum LST during the wet season, suggesting that newly emerged flies preferred areas with higher LST values when it was cold. The non-teneral flies had a negative relationship with median LST in both seasons. Silt content was only important in explaining the occurrence of the teneral flies in the wet season, and the association was negative.

**Fig. 12:**
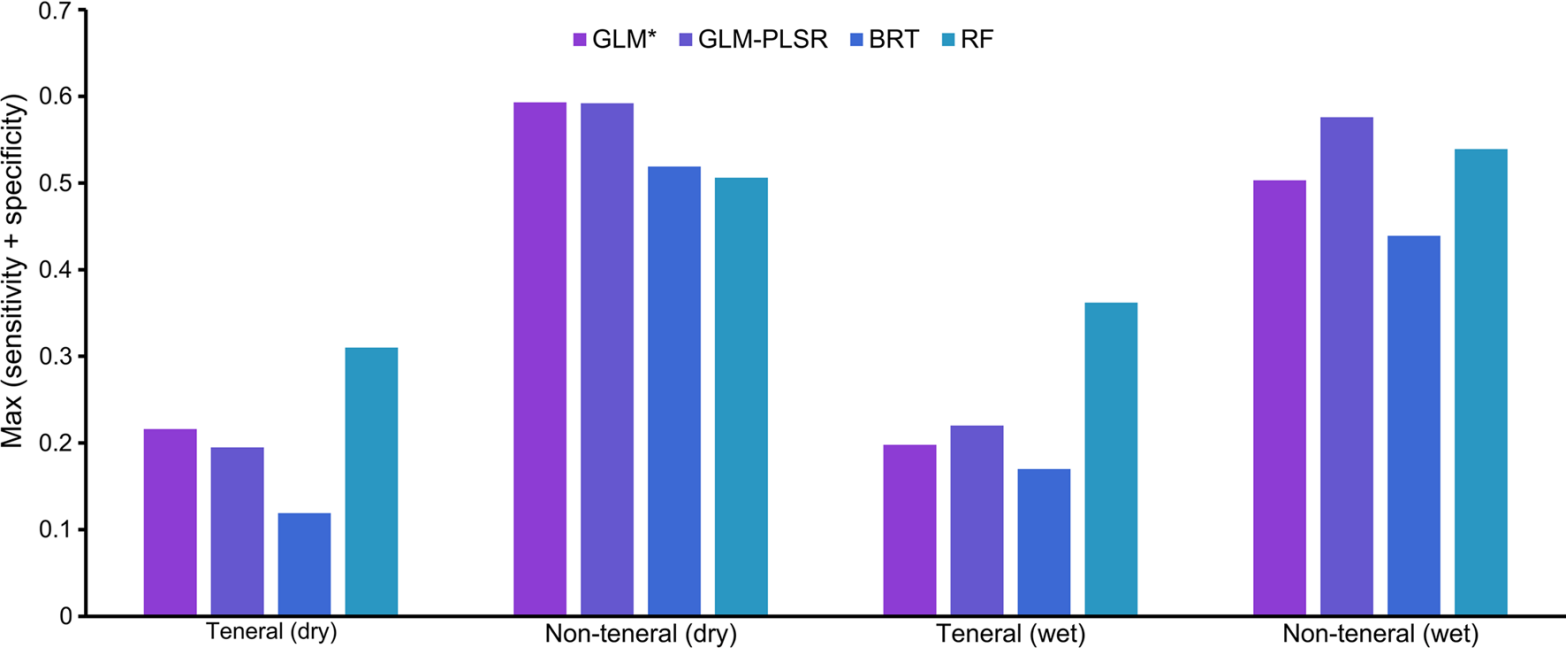
Optimal thresholds that were used to classify suitable and unsuitable teneral and non-teneral areas for the various models

**Fig. 13:**
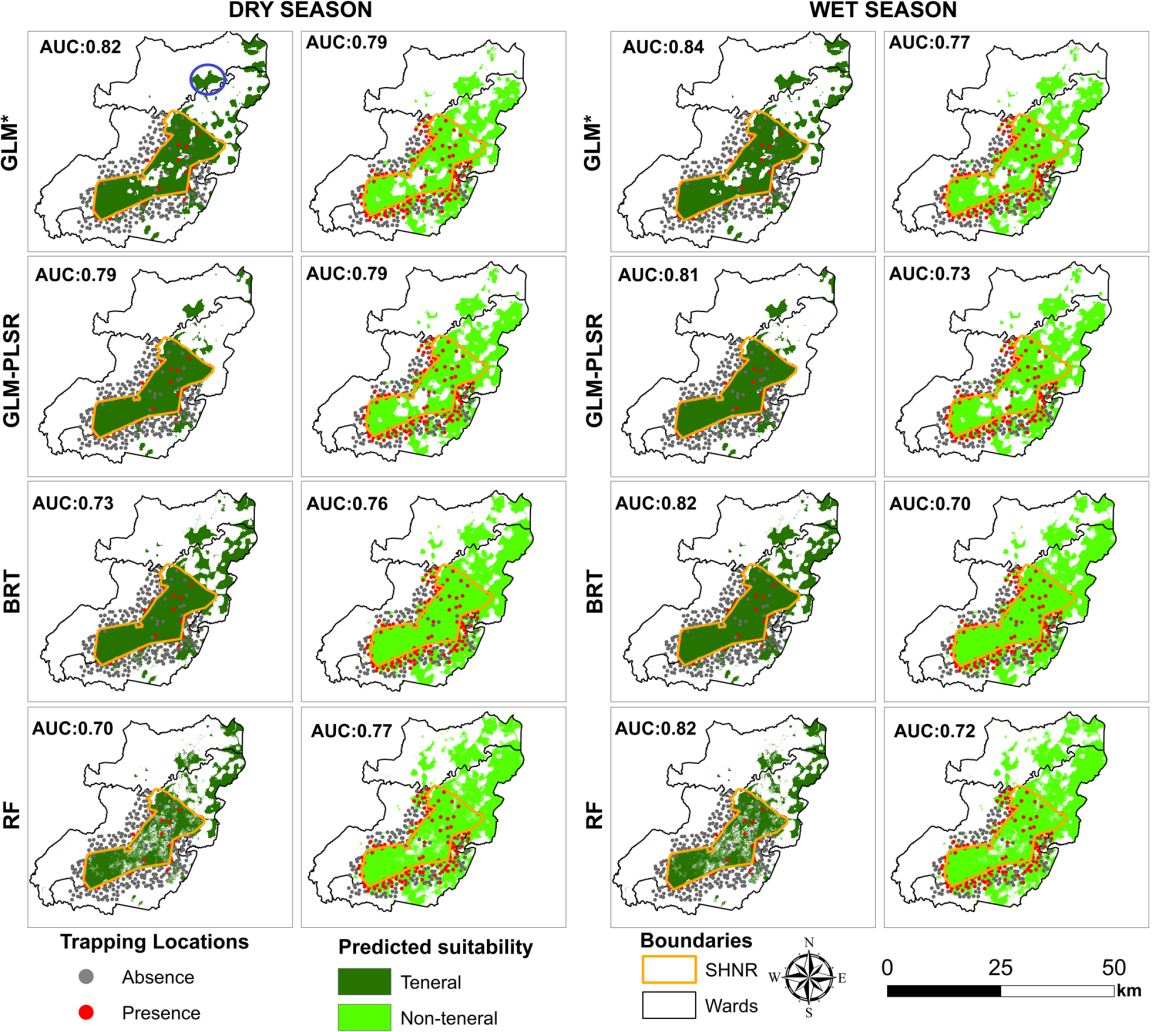
Predicted habitats for the occurrence of the teneral and non-teneral *G. pallidipes* during the dry and wet season with the four different models. The blue circle indicates the Mwaluganje Conservancy

Figure 12 shows the classification thresholds (maximum sum of sensitivity and specificity) that were used to classify the suitable and unsuitable areas. The predicted habitats for the teneral *G. pallidipes* were mostly concentrated inside the reserve. While the non-teneral habitats also occurred in the reserve, they extended far beyond the park boundaries, especially towards the south-eastern side (Fig. 13). All models predicted unsuitable habitats for the occurrence of both the teneral and non-teneral *G. pallidipes* towards the western side of the study area. A prediction of high suitability can be observed for Mwaluganje Conservancy, which is a known tsetse fly hotspot (Fig. 13, blue circle) but for which no observations were included in the current model fitting.

## Discussion

Our results showed that the most important variables for predicting the occurrence of both life stages were mostly consistent across the models. Among the four models, GLM with a backward stepwise regression model had higher AUC values (Fig. 8) across all life stages, and thus this model result was used for this discussion. In general, teneral *G. pallidipes* occurrence models had higher AUC values than those of the non-teneral flies. This could be because the newly emerged flies that have not had a blood meal are likely to be within more restricted ranges than non-teneral flies that move further in search of hosts to feed on. While some environmental factors were the same for teneral and non-teneral flies, we also found differences that varied between the dry and the wet seasons. Cropland and woodland fractions were important for modelling both teneral and non-teneral flies, but other parameters were only relevant for modelling a single life stage. For instance, grassland fraction and median LST were only important for the non-teneral flies, while silt content was essential to represent the presence of teneral flies. The direction of the relationship between the important variables and the occurrence of each life stage varied between seasons and at times between life stages, other than the cropland fractions, which were negatively correlated with both life phases regardless of the season.

The lack of shading in cropland fractions likely explains the strong negative correlation with both *G. pallidipes* life stages*.* Human-induced environmental changes such as increased land cultivation negatively affect tsetse fly occurrence [45–47]. Previously, intensive bush clearing that aimed to clear shaded areas where tsetse flies laid their larvae and rested was used as a tsetse control strategy, and it did lead to a tremendous decline in tsetse numbers and trypanosomiasis [48]. Shaded areas are important for tsetse breeding sites [5], and this could explain the strong positive relationship between the teneral *G. pallidipes* and the woodland fractions in both seasons.

Other than the need for shaded places to rest [49], adult tsetse require blood meals to survive. Therefore, their distribution is also influenced by that of the hosts they feed on [50]. Although the present study did not include host data, animal distribution is influenced by the seasonal changes in the environment such as vegetation cover changes. For instance, wildlife will likely move towards the available vegetated areas (mostly the woodlands and scattered shrubs/tree crops) during the dry season to find fodder and avoid excessive temperatures. Their presence in these areas implies that a blood source is easily available, and thus may explain the positive relationship between the non-teneral flies and the woodland and scattered shrub fractions in the dry season. During the wet season, potential hosts are more dispersed into the open grasslands, causing flies to disperse further for feeding; this may explain the negative correlation of non-teneral flies with woodlands in the wet season. In addition, given the lower temperatures in the wet season, flies will likely move out of the cooler woodlands to more open/sunlit areas.

Dry external environments or waterlogging can lead to high pupal mortality rates through dryness or drowning [5]. This could explain why during the wet season, teneral flies had a negative relationship with silt content, which is associated with high moisture retention. We initially hypothesized that MNDWI would capture the soil moisture variability and thus be a useful proxy to determine the importance of a moist external environment for the occurrence of teneral flies, but this was not the case. This could be because other environmental factors such as land cover and LST that correlate with soil moisture [51] were already incorporated in the models.

Very low or high temperatures have negative effects on the survival of both young and adult tsetse flies [51–53]. Temperatures that are suboptimal for tsetse occurrence are season- and location-dependent. However, a recent study by Are and Hargrove [54] shows that most tsetse fly species cannot survive below 16 °C or above 32 °C. In this study, we used LST as the temperature indicator. However, vegetation and shading will cause small-scale variability in air temperature, which is not incorporated in the pixel-level LST retrievals. Although Ngonyoka et al. [55] did not find a relationship between *G. pallidipes* abundance and LST for the Maasai Steppe in Tanzania, in this study newly emerged *G. pallidipes* correlated positively with maximum LST during the wet season, while adult flies correlated negatively with median LST in both seasons. The response curve (Figs. 10 and 11) between adult flies and median LST intersected with the optimal thresholds (Fig. 12) used to categorize the suitable and unsuitable areas at ~ 24 °C in both seasons, indicating that the probability of *G. pallidipes* occurrence decreases below these temperatures regardless of the season.

Extrapolation to larger extents resulted in an observable spatial consistency across all models used. The predicted suitability of teneral and non-teneral flies overlapped, but the former was more restricted inside the SHNR, suggesting that breeding predominantly occurs within the reserve boundaries (Fig. 13). The extension of non-teneral habitats from teneral habitats confirms that tsetse fly dispersal involves movements between their home ranges and habitual feeding grounds [7, 55]. While the present study cannot report on the accuracy of prediction when extrapolating beyond the sampled area, the results indicated occurrence in areas known to be suitable for tsetse flies such as the Mwaluganje Conservancy (Fig. 13, blue circle). This possible extrapolation beyond the sampled region can be used to identify potential hotspots of *G. pallidipes* presence and guide in situ monitoring efforts.

Although this study provided insights into the importance of environmental variables for teneral and non-teneral tsetse occurrence, it was limited in that it did not include data on the presence of vertebrate hosts, which is critical for the survival of adult tsetse flies [56]. Additionally, due to the random sampling method used for the tsetse data collection, not all land cover class abundances (a key factor in tsetse occurrence) were equally represented (Additional File 1: Figure S2 and Figure S3). For example, while the woodland and grasslands fractions were well sampled, allowing for robust conclusions on the positive and negative relationships, the importance of other land cover fractions (or lack thereof) is less robustly confirmed in this study. We also transformed tsetse fly trap data from three calendar years (2017–2019) into two seasons (Figs. 2 and  3) but used satellite images that were captured in 2019 to generate the predictor variables that were used in the model fitting. We assumed that environmental changes (especially the land cover) between the 3 years were unlikely to have drastic effects on habitat suitability. Unlike other disease vectors (for example, mosquitos [12]), weather changes have less impact on the occurrence of the tsetse fly. Nonetheless, such weather and environmental temporal effects may need to be addressed when predicting tsetse abundance. An understanding of environmental effects on the apparent tsetse density, and consequently using this information to predict their relative abundance, is likely to provide further insight on areas of high AAT risk as compared to the habitat suitability mapping performed here. Despite these shortcomings, the study highlights that teneral fly trapping data could be used as a proxy for mapping potential breeding sites, while non-teneral fly suitability can be used to assess the dispersal of the adult flies from those breeding areas.

## Conclusion

Modelling the distribution of teneral and non-teneral *G. pallidipes* separately allows us to predict potential breeding and foraging grounds for this species. The large-scale identification of tsetse breeding sites provides information on potential tsetse re-invasion fronts. The extension of non-teneral habitats further away from the teneral habitats can be used to estimate adult flies’ dispersal ranges. This provides insight into the ecology of *G. pallidipes* and can be useful for selecting priority areas for control and piloting of field activities.

## Supplementary Information

**Additional file 1:Figure S1.** Boxplots showing the seasonal 16-day MODIS NDVI within the study area (Shimba Hills National Reserve and its surrounding). **Figure S2** and **Figure S3.** Histograms showing the distribution of traps based on the relative land cover classes abundance generated using the 1010 × 1010 m moving window during the dry and wet season, respectively.

## Data Availability

Tsetse and environmental variables data used in this study are available at the Zenodo data repository center (http://doi.org/10.5281/zenodo.4534415).

## Abbreviations

AAT: Animal African trypanosomiasis
AIC: Akaike information criterion
AUC: Area under the curve
BRT: Boosted regression trees
EVI: Enhanced Vegetation Index
FAO: Food and Agriculture Organization
FTD: Flies per trap per day
GEE: Google Earth Engine
GLM: Generalized linear model
GPS: Global positioning system
HAT: Human African trypanosomiasis
LST: Land surface temperature
LULC: Land use/land cover
MNDWI: Modified Normalized Difference Water Index
MODIS: Moderate Resolution Imaging Spectroradiometer
MSAVI2: Modified Soil-Adjusted Vegetation Index
NDVI: Normalized Difference Vegetation Index
OLI: Operational Land Imager
PLSR: Partial least squares regression
RF: Random forest
SHNR: Shimba Hills National Reserve
SRTM: Shuttle Radar Topography Mission
TSS: True skills statistics
USDA: United States Department of Agriculture
VIF: Variance of inflation

## References

[1] Rogers D, Wint W. Predicted distributions of tsetse in Africa. Rome. 2000. https://kdna.net/168-2011/african-tryps/pdf.files-of-papers/Predicted-distri-of-tsetse.pdf. Accessed 10 Sept 2021.

[2] Ngari NN, Gamba DO, Olet PA, Zhao W, Paone M, Cecchi G (2020). Developing a national atlas to support the progressive control of tsetse-transmitted animal trypanosomosis in Kenya. Parasit Vectors.

[3] Meyer A, Holt HR, Selby R, Guitian J (2016). Past and ongoing tsetse and animal trypanosomiasis control operations in five African countries: a systematic review. PLoS Negl Trop Dis.

[4] Buxton PA (1956). The natural history of tsetse flies. Geogr J.

[5] Lambrecht FL (1964). Aspects of evolution and ecology of tsetse flies and trypanosomiasis in prehistoric African environment. J Afr Hist.

[6] Hargrove JW, Maudlin I, Holmes P, Miles M (2004). Tsetse population dynamics. The trypanosomiases.

[7] Brightwell R, Dransfield RD, Williams BG (1992). Factors affecting seasonal dispersal of the tsetse flies *Glossina**pallidipes* and *G*. *longipennis* (Diptera: Glossinidae) at Nguruman, south–west Kenya. Bull Entomol Res.

[8] Vale GA (1974). New field methods for studying the responses of tsetse flies (Diptera, Glossinidae) to hosts. Bull Entomol Res.

[9] Clausen PH, Adeyemi I, Bauer B, Breoller M, Salchow F, Staak C (1998). Host preferences of tsetse (Diptera: Glossinidae) based on bloodmeal identifications. Med Vet Entomol.

[10] Cailly P, Tran A, Balenghien T, L’Ambert G, Toty C, Ezanno P (2012). A climate-driven abundance model to assess mosquito control strategies. Ecol Modell.

[11] Chaves L, Friberg M, Moji K (2020). Synchrony of globally invasive *Aedes* spp. immature mosquitoes along an urban altitudinal gradient in their native range. Sci Total Environ.

[12] Nosrat C, Altamirano J, Anyamba A, Caldwell JM, Damoah R, Mutuku F (2021). Impact of recent climate extremes on mosquito-borne disease transmission in Kenya. PLoS Negl Trop Dis.

[13] Chaves LF, Valerín Cordero JA, Delgado G, Aguilar-Avendaño C, Maynes E, Gutiérrez Alvarado JM (2021). Modeling the association between *Aedes aegypti* ovitrap egg counts, multi-scale remotely sensed environmental data and arboviral cases at Puntarenas, Costa Rica (2017–2018). Curr Res Parasitol Vector Borne Dis.

[14] Tran A, Herbreteau V, Demarchi M, Mangeas M, Roux E, Degenne P, et al. Spatial modeling of mosquito population dynamics: an operational tool for the surveillance of vector-borne diseases. ISRSE-37. 2017. https://agris.fao.org/agris-search/search.do?recordID=FR2019175482. Accessed 16 Nov 2020.

[15] Tran A, Fall AG, Biteye B, Ciss M, Gimonneau G, Castets M (2019). Spatial modeling of mosquito vectors for Rift Valley Fever virus in Northern Senegal: integrating satellite-derived meteorological estimates in population dynamics models. Remote Sens.

[16] Stanton MC, Esterhuizen J, Tirados I, Betts H, Torr SJ (2018). The development of high resolution maps of tsetse abundance to guide interventions against human African trypanosomiasis in northern Uganda. Parasit Vectors.

[17] Diarra B, Diarra M, Diall O, Bass B, Sanogo Y, Coulibaly E (2019). A national atlas of tsetse and African animal trypanosomosis in Mali. Parasit Vectors.

[18] Challier A (1982). The ecology of tsetse (*Glossina* spp.) (Diptera, Glossinidae) a review (1970–1981). Int J Trop Insect Sci.

[19] Fuentes A. Colors of Doom: What does the tsetse fly see?. 2017. https://socobilldurham.stanford.edu/sites/g/files/sbiybj10241/f/final_fuentes_colorsofdoom_sophomorecollege2017.pdf. Accessed 26 May 2020.

[20] Diallo M, Dia I, Diallo D, Diagne CT, Ba Y, Yactayo S (2016). Perspectives and challenges in entomological risk assessment and vector control of chikungunya. J Infect Dis.

[21] Saini RK, Orindi BO, Mbahin N, Andoke JA, Muasa PN, Mbuvi DM (2017). Protecting cows in small holder farms in East Africa from tsetse flies by mimicking the odor profile of a non-host bovid. PLoS Negl Trop Dis.

[22] Kulohoma BW, Wamwenje SAO, Wangwe II, Masila N, Mirieri CK, Wambua L (2020). Prevalence of trypanosomes associated with drug resistance in Shimba Hills, Kwale County, Kenya. BMC Res Notes.

[23] Peel MC, Finlayson BL, McMahon TA (2007). Updated world map of the Köppen-Geiger climate classification. Hydrol Earth Syst Sci.

[24] Luo D, Jin H, Marchenko SS, Romanovsky VE (2018). Difference between near-surface air, land surface and ground surface temperatures and their influences on the frozen ground on the Qinghai–Tibet Plateau. Geoderma.

[25] Jiménez-Muñoz JC, Sobrino JA, Skoković S, Mattar C, Cristóbal J (2014). Land surface temperature retrieval methods from landsat-8 thermal infrared sensor data. IEEE Geosci Remote Sens Lett.

[26] Sørensen R, Zinko U, Seibert J (2006). On the calculation of the topographic wetness index: evaluation of different methods based on field observations. Hydrol Earth Syst Sci.

[27] Zimmerman JL. GIS Topographic Wetness Index (TWI) Exercise Steps. Harrisburg. 2016. https://chesapeakeconservancy.org/wp-content/uploads/2018/12/TWI-Work-Flow-Final.pdf. Accessed 12 Oct 2020.

[28] Salley SW, Herrick JE, Holmes CV, Karl JW, Levi MR, McCord SE (2018). A comparison of soil texture-by-feel estimates: implications for the citizen soil scientist. Soil Sci Soc Am J.

[29] Hengl T, De Jesus JM, Heuvelink GBM, Gonzalez MR, Kilibarda M, Blagotić A (2017). SoilGrids250m: global gridded soil information based on machine learning. PLoS ONE.

[30] Meier M, de Souza E, Francelino MR, Fernandes Filho EI, Schaefer CEGR (2018). Digital soil mapping using machine learning algorithms in a tropical mountainous area. Rev Bras Ciênc Solo.

[31] Quinn GP, Keough MJ, Quinn GP, Keough MJ (2012). Multiple and complex regression. Experimental design and data analysis for biologists.

[32] Shabani F, Kumar L, Ahmadi M (2016). A comparison of absolute performance of different correlative and mechanistic species distribution models in an independent area. Ecol Evol.

[33] McCullagh P, Nelder JA (1989). Generalized linear models.

[34] Friedman JH (2001). Greedy function approximation: a gradient boosting machine. Ann Stat.

[35] Breiman L (2001). Random forests. Mach Learn.

[36] Bertrand F, Maumy-Bertrand M. Partial Least Squares Regression for Generalized Linear Models. 2020. https://github.com/fbertran/plsRglm/. Accessed 21 Aug 2020.

[37] Stoltzfus JC (2011). Logistic regression: a brief primer. Acad Emerg Med.

[38] R Core Team. R: A language and environment for statistical computing. 2020. http://www.r-project.org/index.html. Accessed 21 Aug 2020.

[39] Greenwell B, Boehmke B, Gray B (2020). Variable importance plots—an introduction to the vip package. R J.

[40] Oyedele OF, Lubbe S (2015). The construction of a partial least-squares biplot. J Appl Stat.

[41] Rocha AD, Groen TA, Skidmore AK, Darvishzadeh R, Willemen L (2017). The naïve overfitting index selection (NOIS): a new method to optimize model complexity for hyperspectral data. ISPRS J Photogramm Remote Sens.

[42] Naimi B, Araújo MB (2016). sdm: a reproducible and extensible R platform for species distribution modelling. Ecography.

[43] Liu C, Newell G, White M (2016). On the selection of thresholds for predicting species occurrence with presence-only data. Ecol Evol.

[44] Liu C, Berry PM, Dawson TP, Pearson RG (2005). Selecting thresholds of occurrence in the prediction of species distributions. Ecography.

[45] Hair JF, Sarstedt M, Hopkins L, Kuppelwieser VG (2014). Partial least squares structural equation modeling (PLS-SEM): an emerging tool in business research. Eur Bus Rev.

[46] Bourn D, Reid R, Rogers D, Snow B, Wint W (2001). Environmental change and the autonomous control of tsetse and trypanosomosis in sub-Saharan Africa: case histories from Ethiopia, the Gambia, Kenya, Nigeria and Zimbabwe.

[47] Van den Bossche P, de La RS, Hendrickx G, Bouyer J (2010). A changing environment and the epidemiology of tsetse-transmitted livestock trypanosomiasis. Trends Parasitol.

[48] Kuzoe FAS, Schofield CJ. Strategic review of traps and targets for tsetse and African trypanosomiasis control. 2004. https://www.who.int/tdr/publications/documents/tsetse_traps.pdf. Accessed 22 Oct 2019.

[49] Isherwood F, Duffy B, Resting G (1959). Pallidipes in the Lambwe Valley.

[50] Ducheyne E, Mweempwa C, De Pus C, Vernieuwe H, De Deken R, Hendrickx G (2009). The impact of habitat fragmentation on tsetse abundance on the plateau of eastern Zambia. Prev Vet Med.

[51] Petropoulos G, Barrett B, Petropoulos George (2013). Satellite remote sensing of surface soil moisture. Remote sensing of energy fluxes and soil moisture content.

[52] Hargrove J (2001). The effect of temperature and saturation deficit on mortality in populations of male *Glossina m. morsitans* (Diptera: Glossinidae) in Zimbabwe and Tanzania. Bull Entomol Res.

[53] Krafsur E (2009). Tsetse flies: genetics, evolution, and role as vectors. Infect Genet Evol.

[54] Are EB, Hargrove JW (2020). Extinction probabilities as a function of temperature for populations of tsetse (*Glossina* spp.). PLoS Negl Trop Dis.

[55] Ngonyoka A, Gwakisa PS, Estes AB, Salekwa LP, Nnko HJ, Hudson PJ (2017). Patterns of tsetse abundance and trypanosome infection rates among habitats of surveyed villages in Maasai steppe of northern Tanzania. Infect Dis Poverty.

[56] Muturi CN, Ouma JO, Malele II, Ngure RM, Rutto JJ (2011). Tracking the feeding patterns of tsetse flies (*Glossina* genus) by analysis of bloodmeals using mitochondrial cytochromes genes. PLoS ONE.

[57] Lin S, DeVisser MH, Messina JP (2015). An agent-based model to simulate tsetse fly distribution and control techniques: a case study in Nguruman, Kenya. Ecol Modell.

[58] Xu H (2006). Modification of normalised difference water index (NDWI) to enhance open water features in remotely sensed imagery. Int J Remote Sens.

[59] Chikowore G, Dicko AH, Chinwada P, Zimba M, Shereni W, Roger F (2017). A pilot study to delimit tsetse target populations in Zimbabwe. PLoS Negl Trop Dis.

[60] Lord JS, Hargrove JW, Torr SJ, Vale GA (2018). Climate change and African trypanosomiasis vector populations in Zimbabwe’s Zambezi Valley: a mathematical modelling study. PLOS Med.

[61] Auty H, Morrison LJ, Torr SJ, Lord J (2016). Transmission dynamics of Rhodesian sleeping sickness at the interface of wildlife and livestock areas. Trends Parasitol.

[62] Ersek K. Key Soil Types: Advantages and Disadvantages. HOLGANIX. 2020. https://www.holganix.com/blog/4-key-soil-types-advantages-and-disadvantages. Accessed 16 Aug 2020.

[63] De Risi R, Jalayer F, De Paola F, Lindley S (2018). Delineation of flooding risk hotspots based on digital elevation model, calculated and historical flooding extents: the case of Ouagadougou. Stoch Environ Res Risk Assess.

[64] Fu XT, Zhang LP, Wang Y (2019). Effect of slope length and rainfall intensity on runoff and erosion conversion from laboratory to field. Water Resour.

