# Supplementary material for: Satellite-based modelling of potential tsetse (Glossina pallidipes) breeding and foraging sites using teneral and non-teneral fly occurrence data
Source: Parasit Vectors. 2021 Sep 28;14:506. doi: 10.1186/s13071-021-05017-5 (PMC8479894; doi:10.1186/s13071-021-05017-5)
Supplement: Supplementary file 1 — Additional file 1:Figure S1. Boxplots showing the seasonal 16-day MODIS NDVI within the study area (Shimba Hills National Reserve and its surrounding). Figure S2 and Figure S3. Histograms showing the distribution of traps based on the relative land cover classes abundance generated using the 1010 × 1010 m moving window during the dry and wet season, respectively. [file 13071_2021_5017_MOESM1_ESM.pdf]

Additional Information

Satellite-based modelling of potential tsetse (*Glossina pallidipes*) breeding and foraging sites using teneral and non-teneral fly occurrence data

Stella Gachoki, Thomas Groen, Anton Vrieling, Michael Okal, Andrew Skidmore, and Daniel Masiga

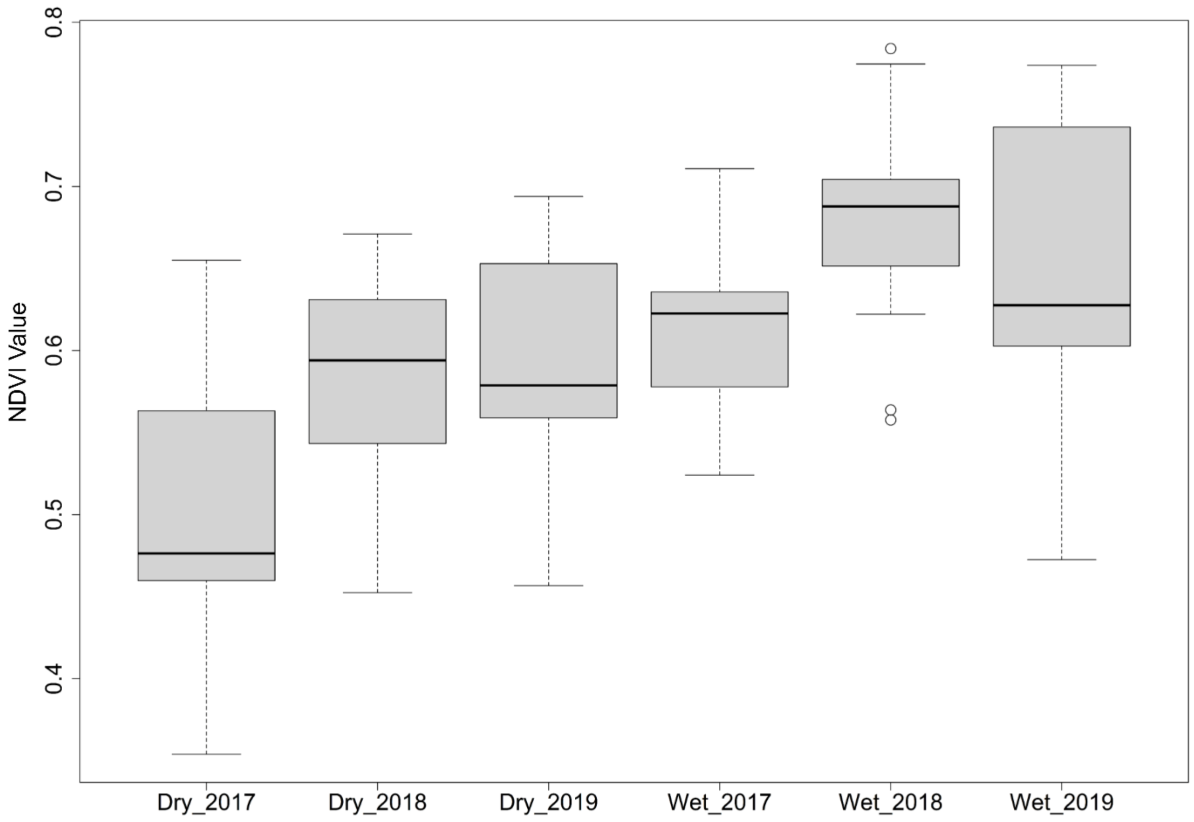

Figure S1. Boxplots showing the seasonal 16-day MODIS NDVI within the study area (Shimba Hills National Reserve and its surrounding).

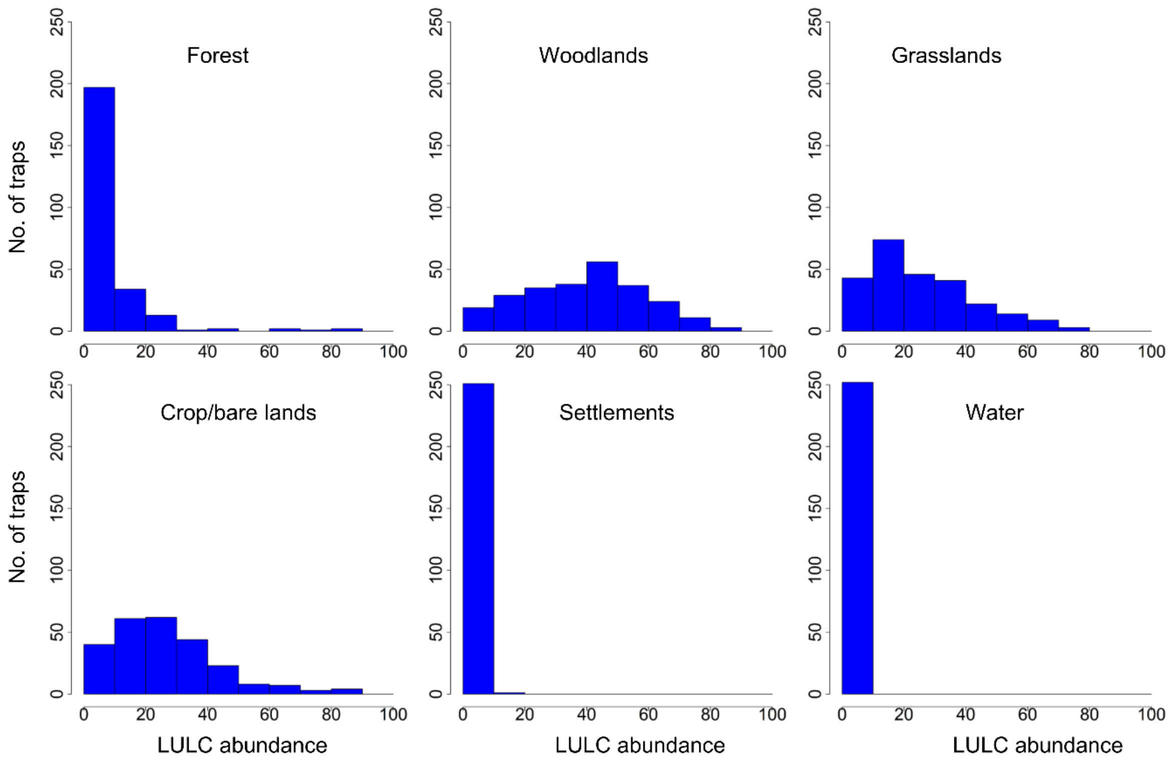

Figure S2. Histograms showing the distribution of traps based on the relative land cover classes abundance generated using the 1010x1010m moving window during the dry season.

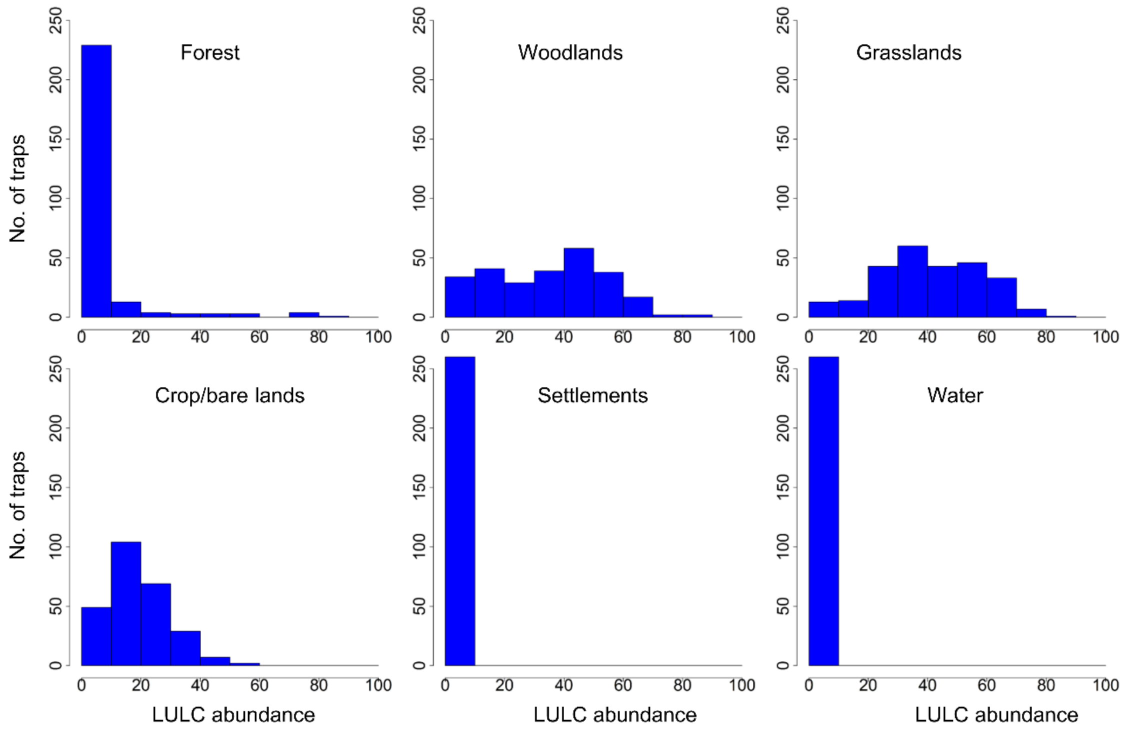

Figure S3. Histograms showing the distribution of traps based on the relative land cover classes abundance generated using the 1010x1010m moving window during the wet season.
